# Amazing Discoveries of Benthic Fauna from the Abyssal Zone of Lake Baikal

**DOI:** 10.3390/biology10100972

**Published:** 2021-09-27

**Authors:** Ilya G. Kondratov, Tatiana Ya. Sitnikova, Irina A. Kaygorodova, Natalia N. Denikina, Vadim V. Annenkov, Igor V. Khanaev, Sergei V. Kirilchik, Ivan A. Nebesnykh, Elena V. Dzyuba

**Affiliations:** Limnological Institute, Siberian Branch of the Russian Academy of Sciences, Ulan-Batorskaya Str. 3, 664033 Irkutsk, Russia; kondratovig@mail.ru (I.G.K.); tata-sitnikova@mail.ru (T.Y.S.); irina@lin.irk.ru (I.A.K.); annenkov@lin.irk.ru (V.V.A.); igkhan@lin.irk.ru (I.V.K.); kirilchik@yandex.ru (S.V.K.); canis-87@mail.ru (I.A.N.); e_dzuba@lin.irk.ru (E.V.D.)

**Keywords:** bryozoans, cocoons of leeches, eggs of gastropods, stramenopiles, COI mtDNA sequences, Lake Baikal

## Abstract

**Simple Summary:**

Lake Baikal is the deepest and oldest freshwater ecosystem in the world. Since more than half of the currently known species of aquatic organisms inhabiting Lake Baikal are endemic, it is called a natural laboratory for the study of species diversity and evolution. However, despite many years of research, the true biodiversity of the lake is clearly insufficiently studied. As before, the deep-water zones of the lake remain white spots: there is little information about small benthic organisms, especially sessile forms. Therefore, when, for the first time, during deep-sea trawls, fragments of packaging plastic were raised from the bottom of Lake Baikal, the main goal was to determine the diversity of aquatic organisms inhabiting it. Analysis of the standard fragment of the mitochondrial genome allowed the obtaining of the first nucleotide data for the endemic Baikal bryozoans *Hislopia placoides*, two new species of leeches (Rhynchobdellida, Piscicolidae) and an unknown representative of stramenopiles that is genetically equidistant from the fungi-like organisms, Oomycetes and Chromulinales (Chrysophyta). Nucleotide data of *H. placoides* indirectly confirmed the hypothesis of the partial origin of Baikal fauna from Tethys derivatives. Thus, the abyssal zone of Baikal is an inexhaustible source of information about biodiversity and the evolution of life on the planet.

**Abstract:**

Lake Baikal is a natural laboratory for the study of species diversity and evolution, as a unique freshwater ecosystem meeting the all of the main criteria of the World Heritage Convention. However, despite many years of research, the true biodiversity of the lake is clearly insufficiently studied, especially that of deep-water benthic sessile organisms. For the first time, plastic waste was raised from depths of 110 to 190 m of Lake Baikal. The aim of this study was to examine the biological community inhabiting the plastic substrate using morphological and molecular genetic analysis. Fragments of plastic packaging materials were densely populated: bryozoans, leeches and their cocoons, capsules of gastropod eggs, and turbellaria cocoons were found. All the data obtained as a result of an analysis of the nucleotide sequences of the standard bar-coding fragment of the mitochondrial genome turned out to be unique. Our results demonstrate the prospects for conducting comprehensive studies of artificial substrates to determine the true biodiversity of benthos in the abyssal zone of Lake Baikal.

## 1. Introduction

Lake Baikal is located in a rift depression on the border of Eastern Siberia and Central Asia and is the deepest lake on the planet (1.642 m) and the largest reservoir of unfrozen surface fresh water (23,000 km^3^). Baikal water is slightly mineralised (the sum of the major ions ~ 96 mg/L) with an unessential content of biogenic elements and high oxygenation (from 11.9 mg/L in the upper layers to 9.5 mg/L at a depth of 1600 m) [1]. These conditions ensured the formation of uniquely rich and diverse biota in the lake. More than half of the currently known species of hydrobionts inhabiting Baikal are endemic. Thus, Lake Baikal is a natural laboratory for the study of species diversity and evolution [2], as a unique freshwater ecosystem meeting the all of the main criteria of the World Heritage Convention [3].

However, despite many years of research, the true biodiversity of the lake is clearly insufficiently studied. To the greatest extent, this statement applies to small-sized deep-sea benthic sessile organisms. The reasons for this are obvious: at present, the collection of samples from great depths is possible only with the help of a dredge or an ichthyological beam trawl. Taking into account the peculiarities of the soils and the bottom relief of Lake Baikal [1], the collection of benthic sessile organisms is very problematic. At the same time, the integrity of the samples suffers; often, when lifting, the bodies of aquatic organisms are frayed by stones, sand, and clay. For the same reason, nucleotide data are absent even for long-described organisms.

In the past decade, scientists have been increasingly recording the consequences of negative anthropogenic impacts on the ecology in the coastal zone of the lake, which has manifested as a change in the dominant species of macrophytes, a huge accumulation of filamentous Zygnematales algae and benthic cyanobacteria, as well as disease and death of the endemic Lubomorskiidae sponges [4,5,6,7,8]. The negative anthropogenic impact on the lake ecosystem is not only due to pollution by industrial and domestic sewage but also a rapid increase in the amount of municipal solid waste, the most obvious component of which is plastic. Products made of polymer materials resistant to degradation and decomposition are the most common type of waste within the recreational areas of the coastal zones of Lake Baikal [9].

Previously, it was shown that human activities often result in the occurrence of new habitats for various groups of invertebrates in aquatic ecosystems [10,11,12]. Invertebrates colonise anthropogenic substrates, including a variety of plastics [13]. Plastic waste carried with storms and currents from the coast of Lake Baikal into its abyssal part has not been previously found.

The collecting of plastic waste during trawling in the abyssal zone of the lake initiated the present study. For the first time, we received a unique material for research: samples of benthic sessile organisms from depths of more than 100 m.

The aim of this study was to examine the biological community inhabiting the plastic substrate using morphological and molecular genetic analysis.

Fragments of plastic packaging materials were densely populated; bryozoans, leeches and their cocoons, capsules of gastropod eggs, and turbellaria cocoons were found. The mitochondrial gene of the first subunit of cytochrome C oxidase (COI), a universal marker widely used in DNA barcoding techniques, was chosen as a genetic marker for identifying biological objects [14,15]. All determined nucleotide sequences were found to be unique.

The research results indicate the need for the monitoring of plastic waste pollution of the lake’s abyssal zones aimed at replenishing knowledge about the biodiversity of the lake using comprehensive methods, as well as determining its impact on the ecology of hydrobionts.

## 2. Materials and Methods

### 2.1. Study Site and Sample Collection

**Figure 1:**
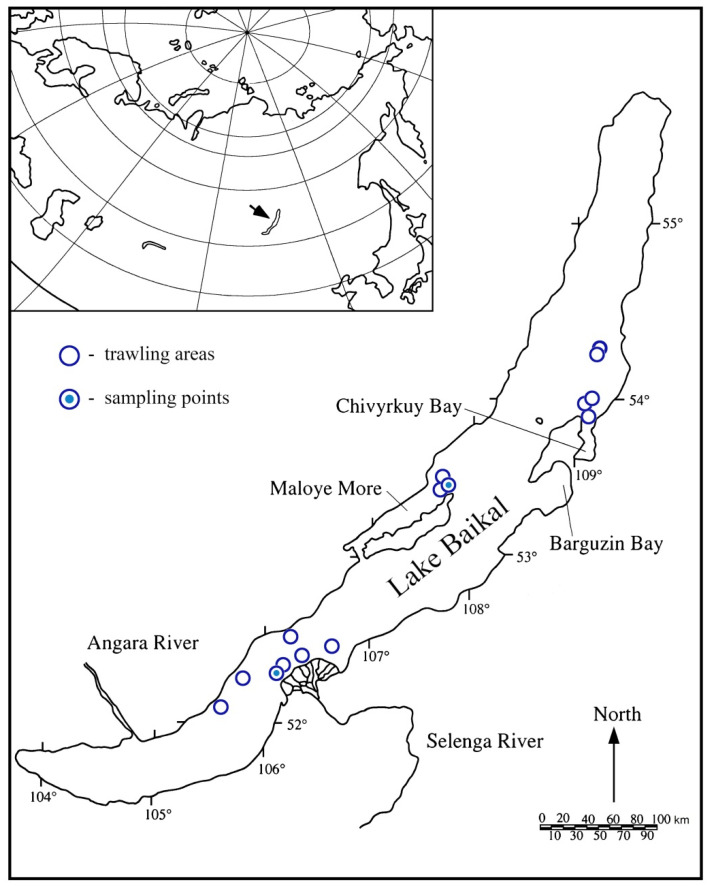
Lake Baikal: trawl map.

Deepwater sampling (depths from 100 to 900 m) was carried out from onboard the research vessel ‘G. Yu. Vereshchagin’ in September 2019. Demersal trawling using an ichthyological beam trawl was repeated 15 times at eight sites of Lake Baikal (Table 1, Figure 1).

Plastic waste was only found at two sites: the area of the Selenga River delta (52°17.270′ N/106°03.910′ E; 52°18.600′ N/106°06.095′ E) at depths of 111 to 133 m, and in the northern section of the Maloye More strait (53°25.280′ N, 107°45.085′ E; 53°24.821′ N, 107°44.079′ E) at depths of 175 to 188 m (Figure 1, sampling points). Amphipods and leeches parasitizing them, planarians, gastropods with their egg capsules on their shell, and cottoid fish were present in the trawls.

**Figure 2:**
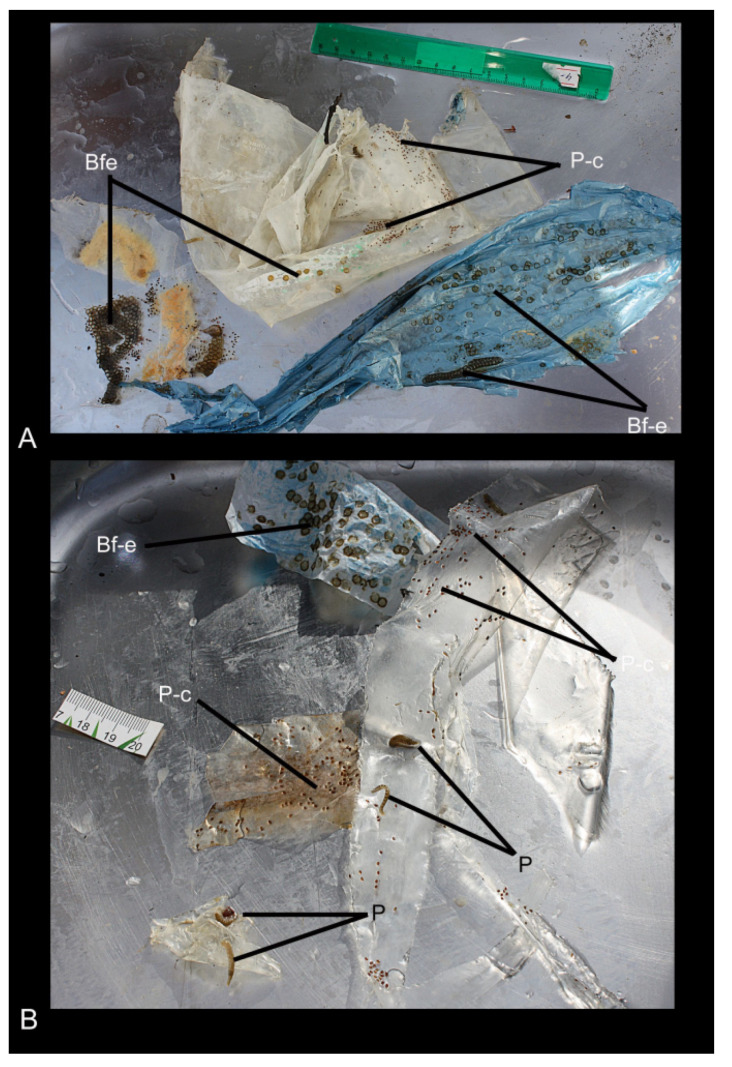
Fragments of packing materials with cocoons of leeches (P-c), leeches (P), and egg capsules (Bf-e) of *Benedictia fragilis* (Dybowski, 1875). (**A**)—from northern region of the Maloye More strait; (**B**)—from the Selenga area.

The waste consisted of five fragments of packing material, colourless, white, and blue plastic, with an area of 0.02 m^2^ to 1.2 m^2^ (Figure 2). During the primary examination, the leeches and bryozoans, the cocoons of the leeches, and the egg capsules of gastropods found on the waste fragments were photographed; the gastropod eggs were immediately identified. The leech cocoons and bryozoans were fixed with 80% ethanol for molecular analyses. Additionally, the leech cocoons were fixed with 4% paraformaldehyde in 0.05% phosphate buffer for morphological study using scanning electron microscopy (SEM). The plastic waste was frozen and transported to the laboratory for further research.

### 2.2. Plastic Composition Experimental Analysis

Fourier-transform infrared spectra (FT-IR) were recorded on an Infralum FT-801 spectrophotometer (SIMEX, Novosibirsk, Russia) in the transmission mode. Before measurement, the films were gently stretched to obtain a thickness that allows spectra with absorption below 1.5 to be obtained. The samples from Lake Baikal were thoroughly washed with SDS solution, then washed with deionised water and dried in a vacuum before the study began. The standard polyethylene and polypropylene spectra were obtained from Aldrich samples (Catalogues Nos. 42805-1 and 45214-9, respectively) cast on the Teflon surface from the melt.

### 2.3. Morphological Analysis

Ten cocoons (5 from the Selenga region and 5 from the Maloye More strait) were rinsed in a liquid of chlorine bleach, washed with distilled water and alcohol, then kept for 12 h in hexamethyldisilazane, according to the method of Saha et al. [16], then dried, gold plated, and examined using SEM (Quanta 200, K. Zeiss).

### 2.4. DNA Analysis

Total DNA was extracted using the DNA-sorb B commercial kit (Research Institute of Epidemiology, Rospotrebnadzor, Moscow, Russia) according to the manufacturer’s protocol.

A cytochrome C oxidase subunit I (COI) gene fragment was amplified using a pair of universal primers, LCO1490/HCO2198 [17], and Phusion High-Fidelity DNA Polymerase (Thermo Fisher Scientific, Carlsbad, CA, USA).

The amplified fragments were cloned into the pJET 1.2 (Fermentas) plasmid vector and analysed by PCR with the recommended pJET1.2 Forward/pJET1.2 Revers primers. The nucleotide sequences of both chains were determined through direct sequencing using the BigDye Terminator v3.1 Cycle Sequencing Kit on a 3500 Genetic Analyzer (Thermo Fisher Scientific), according to the manufacturer’s protocol. All nucleotide sequences were deposited into the GenBank international database under the following numbers: MN854831–MN854837, MN863382–MN863388, and MN866419.

A basic local alignment search [18] was applied to compare the obtained nucleotide sequences of the COI gene fragments with available sequence databases and calculate the statistical significance of the matches. Alignment of the obtained sets of nucleotide sequences was carried out using CLUSTAL W [19].

Visualisations of phylogenetic relationships and calculations of inter- and intragroup genetic distances were implemented using MEGA7.0, the maximum likelihood method [20]. Molecular evolution models were selected using the ModelTest-NG algorithm [21]. The best-fit models for the substitution patterns were GTR+G+I for leeches and Tamura 3-parameter+G+I for bryozoans [22,23].

The phylogenetic analyses of leeches and bryozoans involved 45 and 99 nucleotide sequences, respectively (Table 2 and Table 3). The sequence length of both datasets was 658 bp.

## 3. Results

### 3.1. The Chemical Composition of Plastic Waste

**Figure 3:**
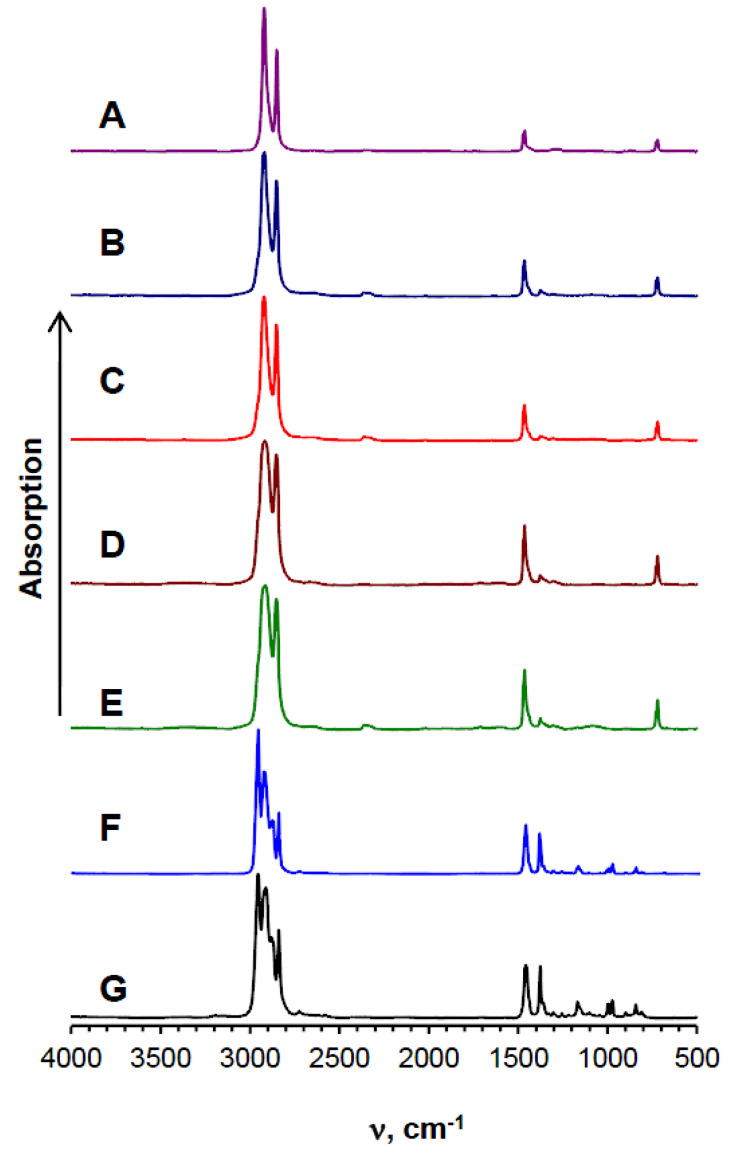
Fourier-transform infrared spectra of standard polyethylene (**A**), colourless (**B**), white (**C**), and blue (**D**) films from the Maloe More strait, colourless (**E**) films from the Selenga area, standard polypropylene (**F**), and a blue film (**G**) from the Selenga area.

FT–IR spectra of the plastic samples collected in the Maloye More strait (Figure 3B–D) and the colourless plastic collected in the Selenga area (Figure 3E) corresponded to the standard spectrum of polyethylene (Figure 3A); the spectra of the blue plastic (Figure 3G) corresponded to the standard polypropylene spectrum (Figure 3F).

### 3.2. Waste-Associated Macroorganisms

**Figure 4:**
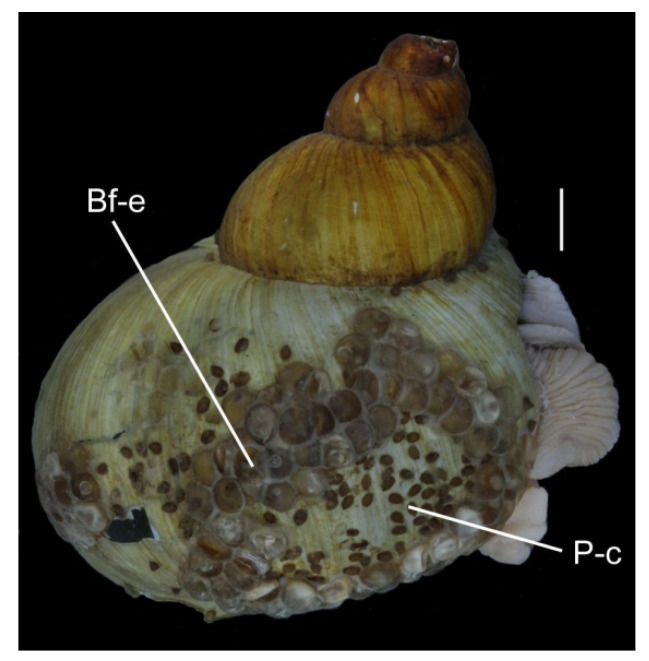
The shell of *Benedictia fragilis* with egg capsules (Bf-e) laid by a snail on the same species and cocoons of leeches (P-c). Scale: 5 mm.

The cocoons of leeches and egg masses of gastropods found were unevenly distributed on the waste fragments (Figure 2); the coverage ranged from 10% (white and colourless plastic) to almost 50% (blue plastic). Some areas of polyethylene fragments of both colours were completely free of eggs. Large egg capsules (approximately 5 mm in diameter) in the form of a smooth hemisphere belonged to the Baikal endemic gastropods, *Benedictia fragilis*, whose adult individuals were collected together with the waste in the trawls. Similar egg capsules and leech cocoons attended the shells of this gastropod species (Figure 4).

On the plastic surface, egg capsules of snails were located both individually and in dense groups. There were 186 capsules on the plastic with an area of 1.2 m^2^. The proportion of capsules with embryos at the first stages of development was approximately 2%; the rest of the capsules were empty.

Leech cocoons were located close to the egg capsules of the snails, as well as at a distance from them, and did not form dense groups; there were from one to three cocoons in an area of 1 cm^2^. The globular cocoons (3 to 5 mm in diameter) of small Turbellaria were empty, dilapidated on the waste.

**Figure 5:**
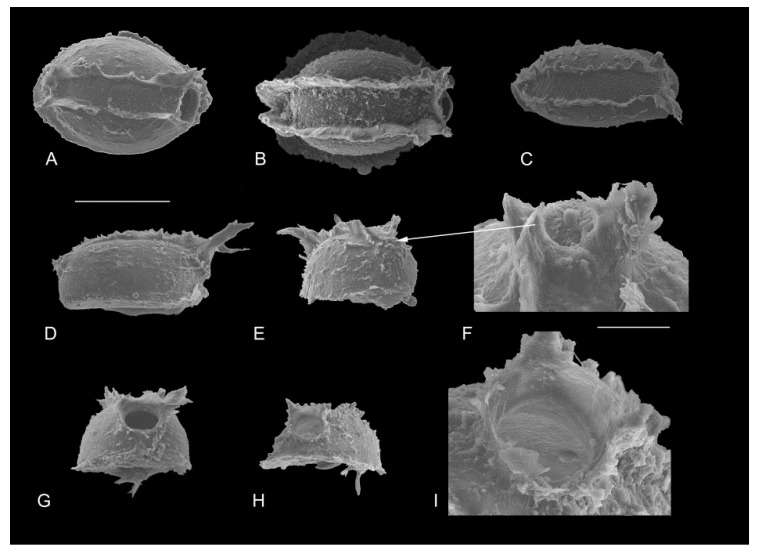
Leech cocoons collected from plastic waste: top view (**A**–**C**); side view (**D**); the back side of the cocoon (**E**,**F**); the front part of the cocoons (**G**–**I**); empty cocoons without operculum (**A**,**G**); cocoons with operculum (**F**,**H**,**I**); enlarged operculum (**I**). Scale: 500 μm (**A**–**E**,**G**,**H**); 100 μm (**F**,**I**).

The cocoons of leeches from the northern region of the Maloye More strait were oblong; their length ranged from 889 μm to 1 mm (1.010 ± 1.175 μm), width—from 430 to 725 μm (594 ± 82), and height—from 247 to 600 μm (434 ± 102). Each cocoon had one aperture covered by an operculum, the diameter of which varied from 115 to 200 μm (168 ± 28; *n* = 10). The dense sheath of the cocoons was sculptured in the form of two parallel protuberances (Figure 5A–C), with the distance between ranging from 175 to 250 μm (216 ± 33; *n* = 8). The round operculum had two protuberances of different lengths; the posterior part of some cocoons had short protuberances (Figure 5F). The cocoons of leeches from the Selenga area had similar sizes and morphologies.

**Figure 6:**
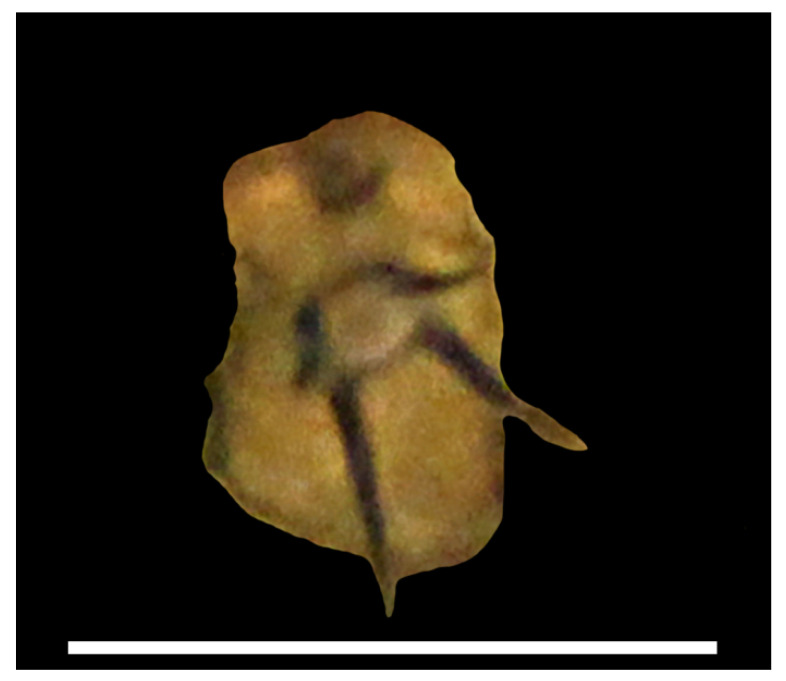
Zooid of bryozoans, *Hislopia placoides* (Korotneff, 1901). Scale: 1 mm.

Bryozoans were found on the polyethylene that was lifted in the Selenga area; some of them were between empty cocoons of leeches. The colonies of bryozoans were short, consisting of three to five zooids with chitin exoskeleton (Figure 6), and similar to *Hislopia placoides* (Korotneff, 1901) in morphology.

### 3.3. Identification of Cocoons

Seven nucleotide sequences of the COI gene fragment (MN854831-MN854837) were obtained from the Maloye More cocoons containing eggs.

Cocoon COI sequences were searched by homology using the BLASTN 2.11.0+ [18], through the NCBI online platform (https://blast.ncbi.nlm.nih.gov; accessed on 26 May 2021). The search found 100 sequences belonging to Rhynchobdellida. The top of the list was formed by 33 different Piscicolidae species with the percent identity ranging between 82.07–90.51%. All of them were included in phylogenetic analysis as a comparison group, whereas representatives of Glossiphoniidae, another Rhynchobdellida family, were used as the outgroup.

**Figure 7:**
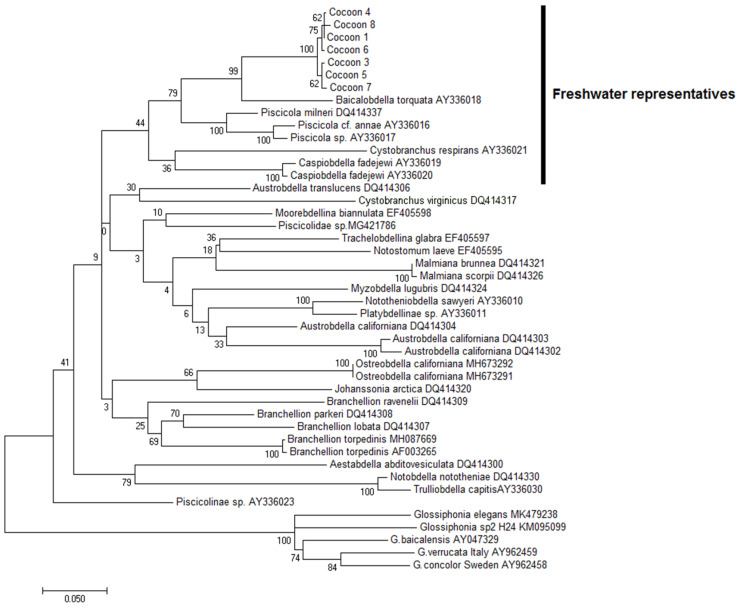
Maximum likelihood phylogenetic tree visualizing the phylogenetic position of the studied leech cocoons.

The phylogenetic tree showed that the group of the leech cocoon sequences form a single lineage within the freshwater representatives of the Piscicolidae branch with a high probability (99–100%) (Figure 7). COI of cocoons formed a separate clade and appeared to be closely related to the Baikal species, *Baicalobdella torquata* (Grube, 1871) (Figure 7).

The genetic polymorphism within the cocoon sequences is 0.42 ± 0.18% of nucleotide substitutions per site. Since these values coincide with the intraspecific differences typical for rhynchobdellid leeches [24], we should conclude that all cocoons most likely belong to the same species. There are 10 variable sites in the set of cocoons sequences: five transitions and five transversions. Some point mutations of the COI gene fragment led to amino acid replacements, including significant ones. Thus, hydrophobic phenylalanine in one of the sequences is replaced by hydrophilic serine. This fact can evidence the genetic plasticity of the test species.

The genetic distance between the cocoons and the *B. torquata* sequence is 9.85 ± 0.9%, which is comparable with the genetic distances between other Piscicolidae species (Table 4). According to the hypothesis underlying the DNA barcoding approach [14], this level of genetic distances is sufficient to conclude that these phylogenetic groups are taxonomically independent and therefore belong to different species. Unfortunately, the international base of publicly available DNA sequences contains a single nucleotide sequence of the COI belonging to the Baikal piscine leech—*B. torquata* (GenBank Accession No. AY336018).

### 3.4. Detection of Stramenopiles

In the associations with the empty leech cocoons from the Selenga River area, we obtained the nucleotide sequence (650 bp) of the COI mtDNA gene belonging to the stramenopiles clade (GenBank Accession MN866419). This sequence is genetically equidistant from both the fungi-like organism *Phytopythium paucipapillatum* Langenhoven, 2017 (SL-2017a strain STE-U7848; GenBank Accession No. KX372747) (Pythiaceae: Peronosporales), with a p-distance of 23%, and *Pedospumella sinomuralis* Boenigk et Grossmann 2016 (GenBank Accession No. KF697349) (Chrysophyta: Chromulinales), with a p-distance of 22%.

### 3.5. Identification of Bryozoans

**Figure 8:**
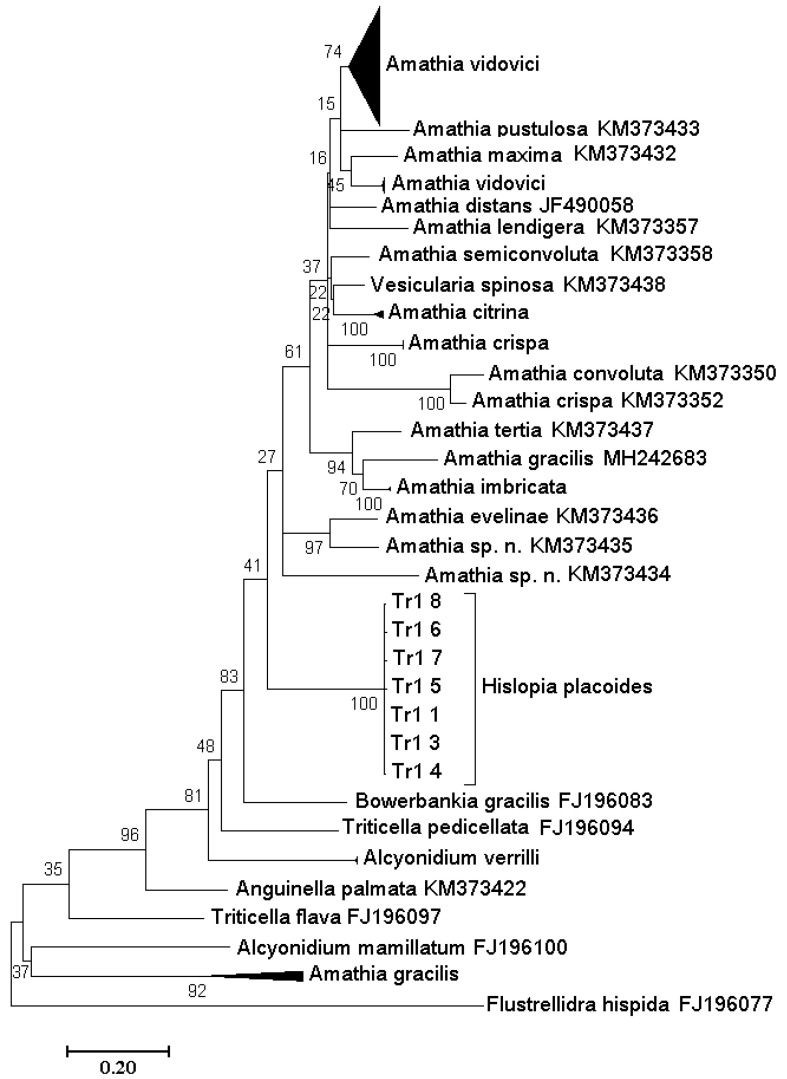
Maximum likelihood phylogenetic tree of Ctenostomatida obtained from 99 nucleotide sequences of COI fragment. Bootstrap values are shown next to the branches (1.000 replicates).

The newly sequenced COI gene fragments from Baikal bryozoans (GenBank Accession Nos.MN863382-MN863388) differed in single-point substitutions (eight transitions and one transversion) that did not lead to significant amino acid replacements. The genetic distances within the group of bryozoans belonging to *H. placoides* sequences were 0.39 ± 0.12%, which, by analogy with the cocoons of leeches, indicates their genetic homogeneity and, as a consequence, their belonging to the same species. The COI sequences of the *H. placoides* clustered in a separate clade (Figure 8) and appeared to be relative of marine bryozoans of the genus *Amathia* Lamouroux, 1812 (Ctenostomatida: Vesiculariidae) (p-distance with *A. imbricata* 20.8 ± 2.0%; *A. vidovici* 20.8 ± 1.9%; *A. distans* 20.8 ± 2.0%) (Table 5).

## 4. Discussion

The presence of plastic waste at two sites of Central Baikal is not accidental, because they are subject to the influence of intense human activity. The Maloye More strait and the Selenga area are the main fishing areas where, before the introduction of the governmental ban on catching the Baikal omul *Coregonus migratorius* (Georgi, 1775) in 2017, commercial fishing was carried out. Nowadays, in addition to illegal fishing and recreational fishing, tourist camps and unorganised tourism contribute to pollution of the lake. Municipal solid waste from the coasts and ice cover of the lake, as well as from its inflowing rivers enters Lake Baikal. The detection of attached forms (bryozoans) and egg clutches (including empty and dilapidated) of benthic hydrobionts on the surfaces of packing materials indicates that the waste has been at the bottom of the lake for quite a long time. Unfortunately, there are no data on the time of hatching from the cocoons of Baikal Piscicolidae. However, earlier, under experimental conditions, it was shown that the hatching time of *Myzobdella lugubris* (Hirudinidae, Piscicolidae) is 48 ± 7 days at 17 °C, and at a temperature of 22 °C, the hatching time is 28 ± 5 days [25]. Taking into account the temperature regime at the bottom of Lake Baikal, we can assume with a fair degree of confidence that the plastic has been there for several months.

Piscine leeches and gastropods are oviparous, needing a substrate for the eggs to attach. However, it is obvious that there is a shortage of solid substrates in the Maloye More strait and in the Selenga area, since the bottom here, especially at the depth zone from 100 to 200 m, is composed of silt and silty sands [26].

The gastropods *B. fragilis*, with large shells (up to 6 cm in height), widespread in Lake Baikal at depths from 30 to 1300 m, are common inhabitants of the silt sands of the lake. In the absence of hard surfaces, they adapted, attaching their eggs to the smooth shell of their own species [27].

To date, there is no information about natural substrates used by the Baikal endemic leeches for attaching cocoons. Nevertheless, freshwater leeches avoid biotopes with soft and unstable substrates such as ooze and sand [11,28]. Piscine leeches usually attach cocoons to aquatic plants, rocky substrates, sunken trees, shells of molluscs, and exoskeletons of crustaceans [29,30,31]. Some species lay cocoons on various artificial substrates that enter aquatic ecosystems as a result of human activity [11,32]. At the same time, the density of cocoons, for example, for the species of the family Erpobdellidae on artificial substrates is several times higher than on natural ones [33]. The presence of cocoons on the *B. fragilis* shells and plastic waste (Figure 1) indicates that the Baikal endemic leeches can use any available solid substrate to attach their cocoons.

In the family Piscicolidae from the Maloye More strait, three species of Baikal endemic leeches were described: *Baicalobdella torquata, B. cottidarum* Dogiel, 1957, and *Codonobdella* sp. [34]. Recently, Matveenko and Kaygorodova [35] suggested the existence of two cryptic species morphologically corresponding to *B. torquata*. Our analysis has revealed that the investigated cocoons likely do not belong to *B. torquata*, whose nucleotide sequences are represented in GenBank. Moreover, representatives of the genus *Baicalobdella* are parasites of littoral amphipods and cottoid fishes; they were found at depths of 3 to 130 m [36]. It can be assumed that the cocoons that we found on the plastic waste could belong to another Baikal leech, *Codonobdella* sp., exploiting deep-water amphipods and ecologically confined to the sublittoral zone of Lake Baikal [34].

Since the samples of cocoons were taken within a depth range from 175 to 188 m, we assume that the polyethylene waste with the cocoons was initially located in the shallow zone of the lake and carried deeper by currents. The same cause can also explain the presence of bryozoans deeper than 150 m, the maximum depth mentioned by Vinogradov [37] for *H. placoides*.

Bryozoans are colonial animals leading a sessile lifestyle. Stones, aquatic plants, sunken wood, shells of molluscs, crayfish shells, and other objects, including plastic, serve as substrates for bryozoans. The experiment has revealed that larvae of the *Bugula* bryozoans choose plastic rather than wooden surfaces [38]. The genetic relationship of *H. placoides* with the marine representatives of Ctenostomatida indirectly confirms the opinion of Pelseneer [39] about their relatively recent divergence. The absence of COI mtDNA sequences of *Hislopia* from other lakes in GenBank does not allow us to clarify the taxonomic status of the Baikal bryozoans with a chitin exoskeleton and explain their origin in Lake Baikal. Large genetic distances between species within Ctenostomatida (Table 4) indicate that these animals are poorly studied. It is noteworthy that the accumulation of information about nucleotide sequences of bryozoans often leads to the identification of novel cryptic species or a redescription of the known ones [40,41]. Since *H. placoides* has been described in four morphotypes inhabiting different depths [36], we can assume that several *Hislopia* species exist in Lake Baikal. Of course, this hypothesis requires further research.

The egg cocoon shells of leeches and gastropods are composed of polysaccharides and fibrous proteins [34,42,43,44] and serve as substrates for colonies of various microorganisms and small fouling organisms. The detected sequence of stramenopiles appeared to be genetically equidistant from the fungi-like organisms Oomycetes and Chromulinales of Chrysophyta, being new for Lake Baikal. The closest relative from oomycetes, *Phytopythium paucipapillatum*, was isolated in South Africa, and may be a soil inhabitant [45]. The genus *Phytopythium* includes more than 20 species, most of which are saprophytic [46]. *Pedospumella sinomuralis* soil colourless Chrysophyta was described in China, 795 m asl [47]. Notably, in the water column of Lake Baikal, stramenopiles were the most diverse group (especially Chrysophyceae), encompassing 562 OTUs of 18S rDNA [48]. According to Yi et al. [49], Baikal microeukaryote diversity is very high and ecologically differentiated. The bottom stramenopiles have not been studied at all in Lake Baikal. The study of the organisms that are not available by conventional collection methods but inhabit plastic waste could lead to unique discoveries.

Despite the fact that our results on the community of benthic organisms associated with plastic waste are geographically limited, they can contribute to the understanding of the mechanisms of the spread of animals in previously uncharacteristic biotopes. At present, this is especially important when studying the distribution of invasive species [50] and the creation of new habitats for animals [51].

The endemic invertebrates effectively use plastic waste to attach cocoons and egg masses. Coming from shallow areas to great depths of Lake Baikal, plastic waste can contribute to the distribution of animals to previously untypical biotopes. The research results indicate the need for the monitoring of plastic waste pollution of the lake’s abyssal zones, aimed at replenishing knowledge about the biodiversity of the lake using comprehensive methods as well as determining the impact of waste pollution on the ecology of hydrobionts. It is necessary to conduct a qualitative and quantitative assessment of micro- and macroeukaryotes inhabiting plastic and an experimental study of the rate of colonisation of organisms and the development of communities of microorganisms, including those capable of destroying plastic.

## Figures and Tables

**Table 1:** Places and coordinates of trawls.

Trawl	Place Name	Coordinates down (N/E)	Coordinates up (N/E)	Depths (m)
1	Selenga River delta	52°17.270′/106°03.910′	52°18.600′/106°06.095′	111 to 133
2	Selenga River delta	52°19.910′/106°07.441′	52°22.280′/106°10.015′	142 to 181
3	Selenga River delta	52°24.933′/106°13.027′	52°26.217′/106°16.940′	271 to 276
4	Proval Bay	52°30.502′/106°36.704′	52°31.196′/106°40.088′	553 to 478
5	Chivyrkuy Bay	53°55.900′/109°08.473′	53°58.341′/109°05.869′	790 to 760
6	Chivyrkuy Bay	53°56.260′/109°06.425′	53°57.765′/109°07.358′	785 to 810
7	Chivyrkuy Bay	53°49.813′/109°08.584′	53°50.787′/109°03.886′	125 to 180
8	Davsha	54°23.662′/109°14.127′	54°23.091′/109°15.727′	700 to 715
9	Davsha	54°21.745′/109°14.749′	54°21.006′/109°12.765′	715 to 715
10	Northern section of the Maloye More strait	53°26.746′/107°44.691′	53°26.496′/107°43.012′	313 to 300
11	Northern section of the Maloye More strait	53°25.280′/107°45.085′	53°24.821′/107°44.079′	175 to 188
12	Northern section of the Maloye More strait	53°24.979′/107°44.783′	53°24.585′/107°43.074′	163 to 186
13	Buguldeyka	52°30.870′/106°07.467′	52°29.179′/106°07.226′	360 to 395
14	Peschanaya	52°15.864′/105°50.365′	52°14.974′/105°49.767′	1005 to 1015
15	Verkhniye Khomuty	52°06.481′/105°37.545′	52°05.767′/105°39.826′	1136 to 1200

**Table 2:** Most relative sequences of Hirudinea selected by BLAST.

Description	Max Score	Query Cover	Per. Ident	Accession
*Baicalobdella torquata*	833	95%	90.51%	AY336018
*Piscicola milneri*	813	99%	88.82%	DQ414337
*Piscicola* cf. *annae*	771	94%	88.71%	AY336016
*Moorebdellina biannulata*	763	99%	87.46%	EF405598
*Piscicola* sp.	745	93%	88.12%	AY336017
*Branchellion parkeri*	725	99%	86.40%	DQ414308
*Austrobdella translucens*	725	99%	86.40%	DQ414306
*Nototheniobdella sawyeri*	708	99%	86.04%	AY336010
*Branchellion torpedinis*	699	98%	85.84%	MH087669
*Caspiobdella fadejewi*	689	94%	86.46%	AY336019
Piscicolidae sp.	688	98%	85.58%	MG421786
*Branchellion torpedinis*	686	97%	85.69%	AF003265
*Caspiobdella fadejewi*	682	94%	86.26%	AY336020
*Cystobranchus respirans*	669	94%	85.90%	AY336021
*Trachelobdellina glabra*	647	99%	84.31%	EF405597
*Branchellion lobata*	636	99%	83.99%	DQ414307
*Aestabdella abditovesiculata*	636	99%	84.08%	DQ414300
Piscicolinae sp.	634	97%	84.36%	AY336023
*Myzobdella lugubris*	630	99%	83.91%	DQ414324
*Austrobdella californiana*	630	99%	83.86%	DQ414304
*Johanssonia arctica*	625	99%	83.71%	DQ414320
*Cystobranchus virginicus*	625	99%	83.69%	DQ414317
*Branchellion ravenelii*	623	98%	83.76%	DQ414309
*Notostomum laeve*	619	99%	83.56%	EF405595
*Ostreobdella californiana*	616	97%	83.72%	MH673292
*Ostreobdella californiana*	616	97%	83.72%	MH673291
*Malmiana brunnea*	597	99%	82.98%	DQ414321
*Austrobdella californiana*	597	99%	82.96%	DQ414303
Platybdellinae sp.	597	84%	85.66%	AY336011
*Malmiana scorpii*	586	99%	82.68%	DQ414326
*Notobdella nototheniae*	579	98%	82.60%	DQ414330
*Austrobdella californiana*	564	94%	82.93%	DQ414302
*Trulliobdella capitis*	556	98%	82.07%	AY336030
*Glossiphonia elegans*	555	98%	82.00%	MK479238
*Glossiphonia complanata*	527	98%	81.27%	JQ821635

**Table 3:** Most relative sequences of Bryozoa: Ctenostomatida selected by BLAST (Max Score > 460).

Description	Max Score	Query Cover	Per. Ident	Accession
*Amathia evelinae*	601	99%	83.41%	KM373436
*Amathia vidovici*	601	99%	83.36%	KM373394
*Amathia* cf. *vidovici*	601	99%	83.36%	JF490059
*Amathia distans*	601	99%	83.28%	JF490058
*Amathia imbricata*	599	99%	83.16%	KM373430
*Amathia vidovici*	595	99%	83.21%	KM373419
*Amathia vidovici*	595	99%	83.21%	KM373415
*Amathia vidovici*	595	99%	83.21%	KM373414
*Amathia vidovici*	595	99%	83.21%	KM373413
*Amathia vidovici*	595	99%	83.21%	KM373412
*Amathia vidovici*	595	99%	83.21%	KM373411
*Amathia vidovici*	595	99%	83.21%	KM373410
*Amathia vidovici*	595	99%	83.21%	KM373406
*Amathia vidovici*	595	99%	83.21%	KM373404
*Amathia vidovici*	595	99%	83.21%	KM373401
*Amathia vidovici*	595	99%	83.21%	KM373399
*Amathia vidovici*	595	99%	83.21%	KM373395
*Amathia vidovici*	595	99%	83.21%	KM373379
*Amathia vidovici*	595	99%	83.21%	KM373368
*Amathia vidovici*	595	99%	83.21%	KM373367
*Amathia vidovici*	595	99%	83.21%	KM373366
*Amathia vidovici*	593	98%	83.26%	KM373370
*Amathia vidovici*	588	98%	83.05%	KM373421
*Amathia vidovici*	588	96%	83.41%	KM373418
*Amathia vidovici*	588	97%	83.26%	KM373396
*Amathia vidovici*	588	98%	83.05%	KM373380
*Amathia vidovici*	588	97%	83.26%	KM373374
*Amathia vidovici*	588	97%	83.26%	KM373373
*Amathia vidovici*	588	97%	83.26%	KM373372
*Amathia vidovici*	588	97%	83.26%	KM373369
*Amathia vidovici*	588	98%	83.05%	KM373365
*Amathia vidovici*	588	98%	83.05%	KM373364
*Amathia vidovici*	584	97%	83.20%	KM373417
*Amathia vidovici*	584	97%	83.10%	KM373400
*Amathia imbricata*	580	99%	82.55%	KM373431
*Amathia vidovici*	580	96%	83.23%	KM373397
*Amathia vidovici*	579	96%	83.20%	KM373362
*Amathia vidovici*	579	96%	83.20%	KM373361
*Amathia vidovici*	577	95%	83.25%	KM373420
*Amathia vidovici*	577	96%	83.12%	KM373382
*Amathia vidovici*	577	96%	83.12%	KM373381
*Amathia vidovici*	577	96%	83.12%	KM373376
*Amathia vidovici*	577	96%	83.12%	KM373375
*Amathia vidovici*	577	95%	83.25%	KM373371
*Amathia maxima*	571	98%	82.72%	KM373432
*Amathia vidovici*	571	96%	82.92%	KM373402
*Amathia vidovici*	571	96%	82.97%	KM373384
*Amathia vidovici*	571	96%	82.94%	KM373359
*Amathia vidovici*	566	95%	82.97%	KM373378
*Amathia vidovici*	538	95%	81.86%	KM373377
*Bowerbankia gracilis*	518	98%	81.25%	FJ196083
*Amathia vidovici*	510	71%	86.20%	KM373407
*Amathia vidovici*	510	71%	86.20%	KM373405
*Anguinella palmata*	501	98%	80.79%	JN680973
*Amathia vidovici*	499	71%	85.77%	KM373393
*Amathia vidovici*	499	71%	85.77%	KM373391
*Amathia vidovici*	499	71%	85.77%	KM373360
*Amathia vidovici*	492	95%	80.57%	KM373383
*Anguinella palmata*	464	98%	79.69%	KM373422

**Table 4:** Estimates of evolutionary divergence over sequence pairs between cocoons group and nearest related sequences. Standard error estimates are shown above the diagonal.

Cocoons		0.014	0.014	0.013	0.014
*Piscicola* cf. *annae* AY336016	0.11641		0.006	0.015	0.010
*Piscicola* sp. AY336017	0.12425	0.023		0.015	0.009
*Baicalobdella torquata* AY336018	0.09859	0.123	0.129		0.014
*Piscicola milneri* DQ414337	0.11676	0.059	0.052	0.115	

**Table 5:** Estimates of evolutionary divergence over sequence pairs between *Hislopia placoides* group and other representatives Ctenostomatida.

*Hislopia placoides*		0.019	0.022	0.020	0.022	0.019	0.023	0.021	0.020	0.030	0.023	0.023	0.022	0.024	0.022	0.025	0.027	0.025	0.032	0.030	0.022	0.022	0.039
*Amathia vidovici* *2 group*	0.208		0.013	0.012	0.017	0.017	0.014	0.014	0.017	0.030	0.015	0.016	0.014	0.024	0.014	0.015	0.020	0.025	0.033	0.030	0.022	0.021	0.038
*Amathia vidovici* *1 group*	0.236	0.115		0.016	0.020	0.017	0.016	0.015	0.020	0.032	0.016	0.019	0.017	0.025	0.015	0.017	0.022	0.028	0.038	0.036	0.025	0.023	0.039
*Amathia distans*	0.208	0.111	0.136		0.019	0.017	0.017	0.016	0.017	0.033	0.015	0.016	0.014	0.025	0.015	0.017	0.021	0.024	0.038	0.033	0.023	0.022	0.040
*Amathia tertia*	0.237	0.175	0.203	0.179		0.020	0.019	0.020	0.015	0.029	0.016	0.019	0.016	0.027	0.018	0.020	0.023	0.025	0.032	0.032	0.024	0.020	0.043
*Amathia evelinae*	0.217	0.192	0.187	0.190	0.220		0.020	0.019	0.019	0.030	0.019	0.021	0.019	0.023	0.018	0.022	0.022	0.022	0.035	0.032	0.023	0.020	0.036
*Amathia pustulosa*	0.244	0.127	0.144	0.161	0.187	0.234		0.017	0.019	0.029	0.015	0.017	0.017	0.026	0.017	0.019	0.020	0.027	0.031	0.032	0.025	0.024	0.037
*Amathia maxima*	0.224	0.127	0.121	0.140	0.193	0.205	0.153		0.020	0.030	0.016	0.019	0.015	0.026	0.016	0.019	0.021	0.026	0.037	0.032	0.023	0.021	0.043
*Amathia imbricata*	0.208	0.169	0.198	0.166	0.125	0.211	0.194	0.196		0.031	0.017	0.018	0.018	0.025	0.016	0.020	0.022	0.024	0.034	0.034	0.024	0.024	0.040
*Amathia gracilis*	0.368	0.393	0.395	0.408	0.357	0.389	0.377	0.380	0.390		0.030	0.031	0.030	0.027	0.032	0.031	0.026	0.030	0.027	0.027	0.028	0.030	0.030
*Amathia citrina*	0.243	0.144	0.150	0.135	0.165	0.215	0.132	0.140	0.177	0.389		0.016	0.014	0.026	0.014	0.016	0.020	0.026	0.035	0.032	0.024	0.022	0.043
*Amathia crispa*	0.238	0.154	0.181	0.153	0.185	0.229	0.157	0.178	0.180	0.392	0.143		0.017	0.027	0.017	0.020	0.021	0.026	0.037	0.034	0.024	0.023	0.040
*Vesicularia spinosa*	0.235	0.127	0.143	0.128	0.154	0.206	0.164	0.120	0.162	0.368	0.123	0.156		0.026	0.013	0.017	0.019	0.024	0.034	0.032	0.022	0.022	0.041
*Anguinella palmata*	0.259	0.271	0.272	0.268	0.299	0.272	0.284	0.272	0.259	0.345	0.288	0.305	0.263		0.026	0.027	0.028	0.025	0.029	0.026	0.024	0.026	0.035
*Amathia* *semiconvoluta*	0.224	0.125	0.118	0.123	0.175	0.190	0.154	0.130	0.153	0.398	0.119	0.153	0.109	0.280		0.017	0.020	0.024	0.036	0.034	0.025	0.021	0.042
*Amathia lendigera*	0.261	0.143	0.164	0.157	0.206	0.230	0.182	0.177	0.197	0.381	0.148	0.190	0.163	0.280	0.156		0.022	0.025	0.037	0.034	0.027	0.024	0.041
*Amathia convoluta*	0.310	0.210	0.232	0.213	0.253	0.274	0.214	0.226	0.244	0.342	0.209	0.231	0.189	0.313	0.210	0.230		0.026	0.027	0.029	0.026	0.024	0.036
*Alcyonidium* *verrilli*	0.265	0.276	0.297	0.262	0.281	0.255	0.285	0.264	0.269	0.379	0.281	0.281	0.244	0.275	0.252	0.265	0.312		0.034	0.031	0.026	0.025	0.036
*Alcyonidium* *mamillatum*	0.365	0.394	0.419	0.420	0.375	0.412	0.350	0.409	0.402	0.336	0.388	0.416	0.380	0.320	0.406	0.400	0.318	0.399		0.029	0.034	0.030	0.036
*Triticella flava*	0.350	0.357	0.398	0.375	0.362	0.366	0.372	0.353	0.373	0.323	0.360	0.388	0.360	0.266	0.379	0.386	0.335	0.349	0.331		0.026	0.031	0.033
*Triticella* *pedicellata*	0.245	0.242	0.270	0.245	0.244	0.257	0.266	0.240	0.263	0.355	0.259	0.268	0.233	0.256	0.266	0.284	0.293	0.274	0.377	0.266		0.022	0.037
*Bowerbankia* *gracilis*	0.239	0.210	0.234	0.215	0.209	0.220	0.239	0.207	0.235	0.375	0.225	0.251	0.223	0.291	0.215	0.243	0.252	0.256	0.355	0.336	0.233		0.035
*Flustrellidra hispida*	0.481	0.463	0.454	0.464	0.490	0.446	0.435	0.479	0.493	0.392	0.511	0.471	0.481	0.429	0.478	0.469	0.448	0.416	0.419	0.390	0.447	0.412	

## Data Availability

The data on the obtained nucleotide sequences are available at the GenBank (MN854831-MN854837, MN863382-MN863388 and MN866419).
